# Inter-method reliability of paper surveys and computer assisted telephone interviews in a randomized controlled trial of yoga for low back pain

**DOI:** 10.1186/1756-0500-7-227

**Published:** 2014-04-09

**Authors:** Christian J Cerrada, Janice Weinberg, Karen J Sherman, Robert B Saper

**Affiliations:** 1 Department of Family Medicine, Boston University School of Medicine and Boston Medical Center, 1 Boston Medical Center Place, Dowling 5 South, Boston, MA 02118, USA; 2 Department of Biostatistics, Boston University School of Public Health, Boston, MA 02118, USA; 3 Group Health Research Institute, Group Health Cooperative, Seattle, WA 98112, USA; 4 Department of Epidemiology, University of Washington, Seattle, WA 98195, USA; 5 Department of Preventive Medicine, Keck School of Medicine of the University of Southern California, 2001 N Soto Street, 3rd Floor, Los Angeles, CA 90032, USA

**Keywords:** Survey methods, Reliability, Back pain, CATI

## Abstract

**Background:**

Little is known about the reliability of different methods of survey administration in low back pain trials. This analysis was designed to determine the reliability of responses to self-administered paper surveys compared to computer assisted telephone interviews (CATI) for the primary outcomes of pain intensity and back-related function, and secondary outcomes of patient satisfaction, SF-36, and global improvement among participants enrolled in a study of yoga for chronic low back pain.

**Results:**

Pain intensity, back-related function, and both physical and mental health components of the SF-36 showed excellent reliability at all three time points; ICC scores ranged from 0.82 to 0.98. Pain medication use showed good reliability; kappa statistics ranged from 0.68 to 0.78. Patient satisfaction had moderate to excellent reliability; ICC scores ranged from 0.40 to 0.86. Global improvement showed poor reliability at 6 weeks (ICC = 0.24) and 12 weeks (ICC = 0.10).

**Conclusion:**

CATI shows excellent reliability for primary outcomes and at least some secondary outcomes when compared to self-administered paper surveys in a low back pain yoga trial. Having two reliable options for data collection may be helpful to increase response rates for core outcomes in back pain trials.

**Trial registration:**

ClinicalTrials.gov: NCT01761617. Date of trial registration: December 4, 2012.

## Background

The self-administered paper survey is a traditional mode of survey administration and data collection in clinical trials [1]. Self-administered paper surveys allow participants to control the pace and order of the questions and provide a level of privacy, which may encourage responders to answer sensitive questions more truthfully [2,3]. Computer assisted telephone interviews (CATI) and other electronic methods of data collection are also common in survey research. They allow for skip logic patterns, immediate data entry, and predefined ranges for responses, all of which may improve data quality [4,5]. CATI and other electronic methods may also help reduce missing responses to questions [6] and boost overall participant response rates by serving as an alternate mode of data collection for targeting non-responders [7].

As methods of survey administration evolve, especially through electronic means, it is increasingly important to consider the inter-method reliability and quality of data collected by each method. The availability of multiple reliable methods of data collection would allow researchers to tailor their survey administration strategy to reach the most participants. While a number of studies have already compared different methods of survey administration, few have focused specifically on low back pain intensity, back-related function, pain medication usage, and health-related quality of life. The purpose of this study is to determine the reliability of responses to traditional self-administered paper surveys and CATI among participants enrolled in a study of yoga for chronic low back pain (LBP).

## Methods

### Study design and setting

This study was part of a larger study of 95 participants enrolled in a randomized dosing trial comparing 12 weeks of once-weekly yoga classes with twice-weekly yoga classes for chronic LBP. Findings from this study suggest that once-weekly yoga classes, supplemented by home practice, are similarly effective as twice-weekly yoga classes for chronic LBP in a predominantly low minority income population [8].

Detailed methods of the parent study are described elsewhere [8]. Briefly, eligibility requirements included being between 18-64 years old, having non-specific LBP lasting longer than 12 weeks, and having English proficiency sufficient to complete both paper and CATI surveys. Recruitment was targeted at community health centers affiliated with a large urban safety-net hospital in order to yield a predominantly minority study population. A 2x2 factorial design was used to generate four treatment groups: once-weekly yoga classes with paper surveys, once-weekly yoga classes with paper surveys and CATI, twice-weekly yoga classes with paper surveys, and twice-weekly yoga classes with paper surveys and CATI.

The Boston University Institutional Review Board and the participating community health centers’ research committees approved the study. Informed consent for the RCT outlined both the 12 week yoga intervention component and the CATI versus paper survey comparison component. All participants consented to both parts of the study.

### Data collection

All study participants completed baseline paper surveys in person at Boston Medical Center and subsequent paper surveys at six and twelve weeks at the community health center where they attended yoga classes. For practical reasons, including staffing constraints and participant burden, only 45 of the 95 participants enrolled in the larger study were randomized to complete a CATI after each paper survey. At each time point, unblinded study staff notified the 45 participants randomized to the CATI group that they would also complete a CATI version of each of their surveys. Staff members blinded to treatment allocation attempted to administer CATI surveys within 48 hours after the in person paper survey. Blinded research staff conducted the CATI via StudyTRAX (ScienceTRAX, Macon, GA), a web-based electronic data capture system [9]. StudyTRAX displayed questionnaire scripts for the interviewers and utilized pre-programmed skip logic for navigating through survey questions. Access to StudyTRAX was granted through unique user logins and passwords. Access to treatment condition information was restricted from blinded staff members. The phrasing of each telephone survey question was kept as similar as possible to the paper survey questions. Participants were asked to try to respond to each question as accurately as possible rather than attempt to reproduce answers to their previous paper survey.

Survey elements included those commonly used in back pain trials [10]. The parent study had two primary outcomes: average low back pain intensity in the previous week on an 11 point numerical scale (0 = ‘no pain’ and 10 = ‘worst possible pain’) [11,12] and back-related function via the modified Roland Morris Disability Questionnaire (RMDQ), a 23 item scale where higher scores indicate worse functional status [13,14]. Secondary outcomes included pain medication use in the last week (yes/no); health-related quality of life measured by the SF-36 [15]; global improvement of back pain on a 7 point numerical scale (0 = ‘extremely worsened’, 3 = ‘no change’, 6 = ‘extremely improved’); and patient satisfaction (5 point Likert scale, 1 = ‘very satisfied’, 2=’somewhat satisfied’, 3 = ‘not satisfied or dissatisfied’, 4=’somewhat dissatisfied’, 5 = ‘very dissatisfied’) [16].

### Data analysis

Participants’ responses from their paper surveys were entered twice by different blinded study staff and compared to verify accuracy of data entry. To measure reliability between paper and CATI data collection methods, we calculated intraclass correlation coefficients [17] to assess reliability for continuous measures. Kappa statistics were calculated to assess reliability for categorical variables (i.e. pain medication use). Only complete pairs of paper and phone responses for each measure at each time point were included in the reliability analyses. Weighted averages were calculated to determine an average ICC or Kappa score for each outcome across all three time points. Means and standard deviations for primary outcomes collected by paper-only and CATI-only were calculated for the 45 participants at each time point using all available data.

## Results

Table 1 shows baseline characteristics for participants randomized to receive the CATI in addition to the paper survey. About three-fourths were women and approximately 70% were non-white. One-third completed high school education or less. Over half had annual household incomes less than $30,000. One-fourth had low back pain for more than 10 years. Characteristics of participants randomized to CATI were similar to those of the entire sample [8].

**Table 1:** Characteristics of 45 adults with chronic low back pain randomized to complete both paper surveys and computer assisted telephone interviews*

	**Paper and CATI (*n*** **= 45)**
**Sociodemographics**	
Mean age, years (SD)	48.0 (10.6)
Female	34 (76)
Race	
White	14 (31)
Black	24 (53)
Other	7 (16)
Hispanic	4 (9)
US born	39 (87)
Education	
High school or less	15 (33)
Beyond high school	30 (67)
Employment	
Unemployed	13 (29)
Disabled	8 (18)
Employed full or part time	20 (44)
Other	4 (9)
Income	
≤$30,000	25 (56)
≥$30,000	17 (38)
Missing	3 (7)
Health Insurance	
Public-funded only	19 (42)
Private	26 (58)
None	0
**Back Pain History**	
Duration of LBP	
<1 year	2 (4)
1-3 years	11 (24)
4-9 years	20 (44)
>10 years	11 (24)
Missing	1 (2)
Mean days of LBP in the last 3 months (SD)	63 (27)
Mean hours/day of LBP (SD)	9 (7)
Mean days cut back activities due to LBP in the last 4 weeks (SD)	12 (9)
Sciatica	15 (33)

*Except where indicated, all results are presented as n (%).

*Abbreviations*: *CATI* computer assisted telephone interview, *SD* standard deviation, *LBP* low back pain.

All but 9 participants completed the in person survey before the CATI at all time points. The majority of participants completed the CATI survey within 48 hours after the in person survey. Twenty completed the CATI within 3-6 days and 4 within 7-11 days of the in person survey for at least one of the time points. Response rates (Table 2) for baseline survey administration were excellent but declined during 6 and 12 weeks. While response rates declined for both survey administration methods, the non-response rate for telephone surveys was greater compared to paper surveys (29% vs.10%). However, comparing responders and non-responders at 12 weeks showed no substantial differences in sociodemographic and baseline low back pain characteristics except for lower income and less frequency of LBP in non-responders (data not shown). For those who completed either survey, however, missing data were rare: completion rates for all 5 outcome measures ranged from 96.1% to 99.6%.

**Table 2:** Response rates for different survey administration methods by time period

**Method of survey administration**	**Baseline *n *** **(%)**	**6 weeks *n *** **(%)**	**12 weeks *n *** **(%)**
Paper (n = 95)	95 (100)	88 (93)	86 (90)
CATI (n = 45)	44 (98)	32 (71)	32 (71)
Both (n = 45)	44 (98)	30 (67)	31 (69)

*Abbreviations*: *CATI* computer assisted telephone interview.

**Figure 1:**
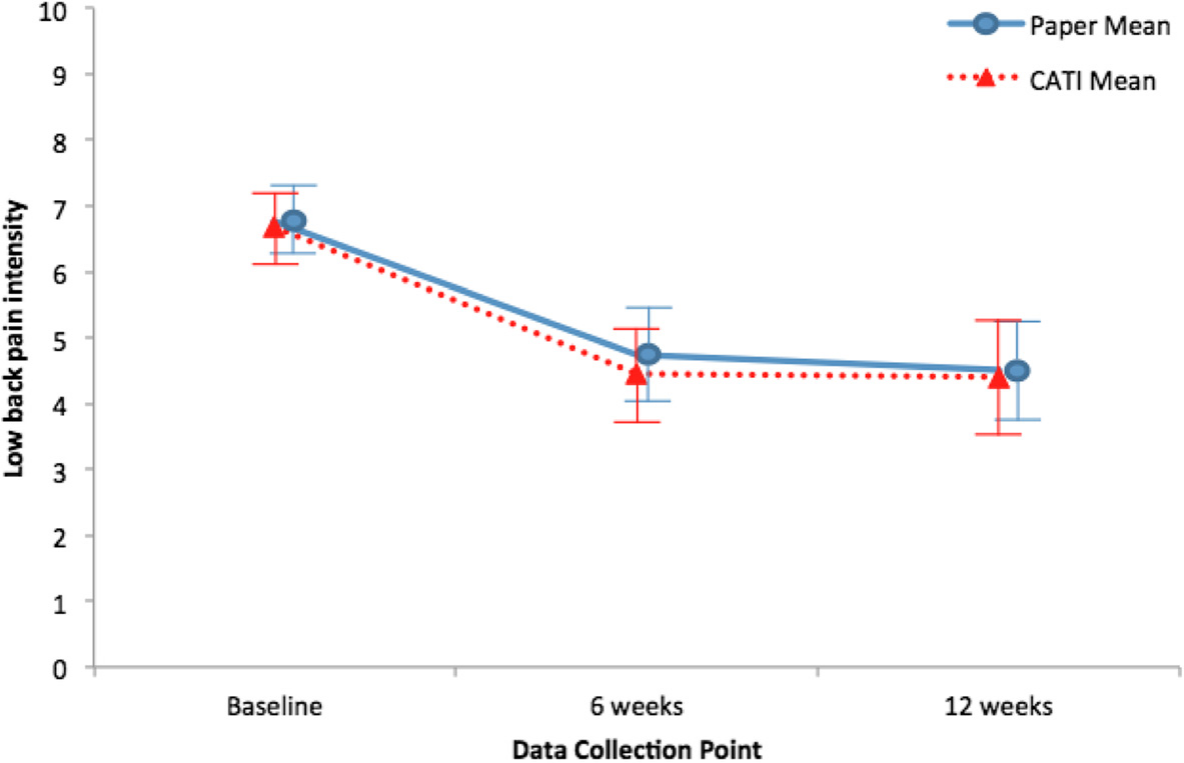
**Mean low back pain intensity over time using all available paper-only data and CATI-only data.** Bars indicate 95% confidence interval. CATI: computer assisted telephone interview.

**Figure 2:**
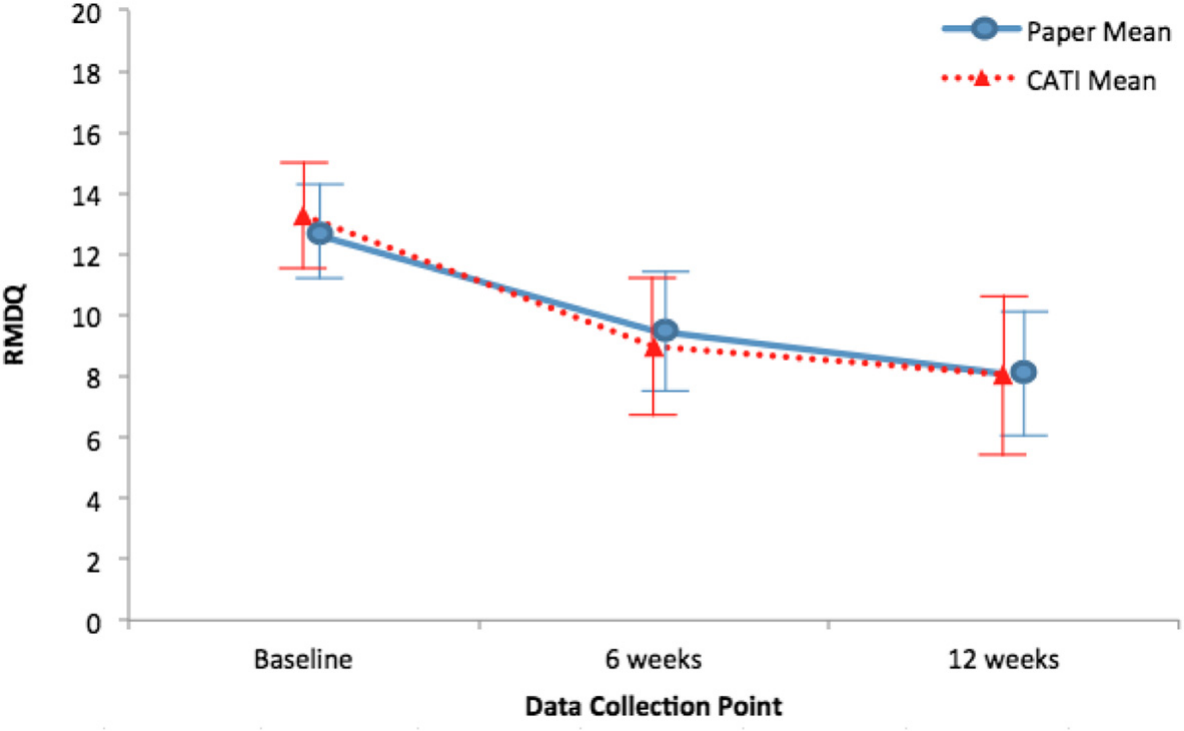
**Mean RMDQ over time using all available paper-only data and CATI-only data.** Bars indicate 95% confidence interval. RMDQ: modified Roland Morris Disability Questionnaire (0-23 where higher scores reflect worse back pain-related function); CATI: computer assisted telephone interview.

Figure 1 shows the low back pain intensity study results derived from CATI data only compared to paper data only using all available data. Similarly, Figure 2 compares RMDQ scores using CATI versus paper data. Both figures demonstrate that study results at all three time points were essentially the same regardless of data collection method. Table 3 shows the intraclass correlation coefficients and kappa statistics for study outcomes. CATIs showed excellent reliability with paper surveys for pain intensity and RMDQ, as evidenced by consistently high intraclass correlations at all three time points. Pain medication use had very good reliability between the two survey methods. Satisfaction had moderate reliability at 6 weeks and excellent reliability at 12 weeks. Both the mental and physical component summary scores for the SF-36 also showed consistently excellent reliability between the two survey methods. Global improvement had poor reliability at all time points.

**Table 3:** Reliability of CATI vs. paper survey administration*

**Measure**	**Baseline**	**6 weeks**	**12 weeks**	**Weighted average**
LBP intensity in the previous week	0.87	0.82	0.92	0.87
RMDQ	0.89	0.92	0.98	0.93
Pain medication use in last week^1^	0.78	0.68	0.72	0.73
Patient satisfaction	-------	0.40	0.86	0.62
Global improvement of back pain	-------	0.24	0.10	0.17
SF-36 Mental Component Summary	0.89	0.86	0.89	0.88
SF-36 Physical Component Summary	0.82	0.82	0.87	0.83

*All reliability statistics are intraclass correlation coefficients except where otherwise noted.

^1^Kappa statistic.

*Abbreviations*: *CATI* computer assisted telephone interview, *LBP* low back pain, *RMDQ* modified Roland Morris Disability Questionnaire, *SF-36* the Short Form-36 Health Survey.

Post-hoc analysis suggests that some participants with discordant responses to global improvement between data collection methods may have misinterpreted the values of the Likert scales. For example, two participants reported ‘extremely worsened’ for global improvement on paper and ‘extremely improved’ on the CATI at 12 weeks. When these two discordant responses were removed from the analysis, the ICC for global improvement increased dramatically from 0.10 to 0.61 at 12 weeks.

## Discussion and conclusion

We compared the inter-method reliability of responses collected by CATI with those collected by a traditional paper method in a study of yoga for chronic LBP. For pain intensity, back-related function, pain medication use, and both physical and mental health components of the SF-36, reliability between paper survey and CATI data collection methods was very good to excellent. Satisfaction with treatment demonstrated moderate reliability at 6 weeks and improved at 12 weeks, whereas global improvement demonstrated poor reliability at every time point. While previous studies have compared the inter-method reliability of paper and telephone interviews for a number of health behavior questionnaires and the SF-36, our study is the first, to our knowledge, to focus on LBP-specific outcome measures such as LBP pain intensity, back-related function (RMDQ), and pain medication use.

The outcomes with greatest inter-method reliability, average low back pain intensity in the past week, back-related function, and pain medication use, are consistent with previous reliability studies [6,18,19]. Pain intensity was measured on a numerical scale from 0 to 10 and both the RMDQ and pain medication use questions were dichotomous, consisting of yes or no response choices. While the SF-36 contains multiple response choices for each question, each response choice also includes clear descriptions. In addition to being relatively straightforward, these measures are ubiquitous in clinical medicine and may be more familiar and intuitive to patients.

Some studies suggest that reliability between methods of survey administration may depend on the nature of the questions asked. For example, Lungenhausen et al [6] found within-subject differences in SF-12 mental health scores but not physical health scores, pain intensity, or pain-related disability between CATI and mailed questionnaires. Lower mental health scores were reported for the self-administered surveys when compared to CATI. Similarly, Feveile et al [19] randomized participants to either mailed questionnaires or telephone interviews and found that for self-assessed mental health items such as well-being, self-esteem, depression, and stress, participants reported more positively over the phone. There was no significant difference in responses between different survey modes for physical health and behavior items like smoking habits and medicine use. It appears that participants may respond differently to questions regarding sensitive topics, such as mental health, and report their health more positively when asked by an interviewer over the phone.

The wording of the question with poor reliability scores (global improvement) was relatively more complex than the others. Dillman [20] suggests that participants may have more difficulty remembering and processing a continuum of response choices, which is the case for Likert scales, and are more likely to choose responses at either extremes of the scale. Without visual cues and set descriptors for each response choice, it is plausible that participants mistakenly reversed the numeric values for ‘extremely worsened’ and ‘extremely improved’ when asked via CATI, resulting in lower ICC scores.

Limitations of our study include the relatively small sample size. While the sample size was chosen for practical considerations, it still provided sufficient precision in order to estimate an ICC. For example, at baseline, the estimated low back pain ICC of 0.87 had an estimate standard error of 0.04 while the estimated baseline Roland ICC of 0.89 had an estimate standard error of 0.03. With a non-response rate of about 10% for paper and 29% for the phone surveys at 6 and 12 weeks, response bias is a possible limitation. However, comparisons between responders and non-responders at 12 weeks showed no differences in most sociodemographic and baseline low back pain characteristics. As demonstrated, small sample size and non-responders may also magnify the effect of very discordant responses on the ICC. Given the relatively short time interval between administrations of each survey method, it is possible that participants may have reproduced responses from their paper surveys on the CATI. We are unable to distinguish inter-method reliability from test-retest reliability. As paper surveys were administered before CATI at all time points, we were also unable to assess the potential effect of survey administration order. Finally, because we targeted a predominantly low income minority population, the results may not be generalizable to a population with higher socioeconomic status.

As researchers begin to utilize new methods of data collection, such as Short Message Service (SMS) and internet surveys, future studies are needed to assess their reliability. Additionally, studies should compare cost, staff burden, and response rates of different data collection methods given the target population. For example, studies report that administering CATI may cost two [21] to three [22] times more than self-administered paper surveys per person. Future work might include an analysis of the costs associated with administering CATI with electronic data capturing systems such as StudyTRAX.

In summary, we studied the reliability of traditional paper surveys and CATI for average low back pain intensity, RMDQ, and pain medication use. At all three time points, the two data collection methods yielded similar results. Having both options for data collection available may be helpful in targeting non-responders and improving overall response rates.

## Competing interests

The authors declare that they have no competing interests.

## Authors’ contributions

CC coordinated data collection, managed StudyTRAX, and drafted the manuscript. JW performed the statistical analysis and data interpretation. KS made critical revisions to the manuscript. RB conceived of the study, participated in its design, and helped to revise the manuscript. All authors read and approved the final manuscript.
